# Abnormal Oculomotor Corollary Discharge Signaling as a Trans-diagnostic Mechanism of Psychosis

**DOI:** 10.1093/schbul/sbad180

**Published:** 2024-01-20

**Authors:** Beier Yao, Martin Rolfs, Rachael Slate, Dominic Roberts, Jessica Fattal, Eric D Achtyes, Ivy F Tso, Vaibhav A Diwadkar, Deborah Kashy, Jacqueline Bao, Katharine N Thakkar

**Affiliations:** Schizophrenia and Bipolar Disorder Program, McLean Hospital, Belmont, MA, USA; Department of Psychiatry, Harvard Medical School, Boston, MA, USA; Department of Psychology, Michigan State University, East Lansing, MI, USA; Department of Psychology, Humboldt University, Berlin, Germany; Department of Psychology, Brigham Young University, Provo, UT, USA; Department of Psychology, Michigan State University, East Lansing, MI, USA; Department of Psychology, Northwestern University, Evanston, IL, USA; Cherry Health, Grand Rapids, MI, USA; Department of Psychiatry, Western Michigan University Homer Stryker M.D. School of Medicine, Kalamazoo, MI, USA; Department of Psychiatry and Behavioral Health, The Ohio State University, Columbus, OH, USA; Department of Psychiatry and Behavioral Neuroscience, Wayne State University, Detroit, MI, USA; Department of Psychology, Michigan State University, East Lansing, MI, USA; Department of Psychology, Michigan State University, East Lansing, MI, USA; Department of Psychology & Neuroscience, Duke University, Durham, NC, USA; Department of Psychology, Michigan State University, East Lansing, MI, USA; Department of Psychiatry and Behavioral Medicine, Michigan State University College of Human Medicine, Grand Rapids, MI, USA

**Keywords:** anomalous self-experiences, eye movements, agency, trans-saccadic perception, schizophrenia, bipolar disorder

## Abstract

**Background and Hypothesis:**

Corollary discharge (CD) signals are “copies” of motor signals sent to sensory areas to predict the corresponding input. They are a posited mechanism enabling one to distinguish actions generated by oneself vs external forces. Consequently, altered CD is a hypothesized mechanism for agency disturbances in psychosis. Previous studies have shown a decreased influence of CD signals on visual perception in individuals with schizophrenia—particularly in those with more severe positive symptoms. We therefore hypothesized that altered CD may be a trans-diagnostic mechanism of psychosis.

**Study Design:**

We examined oculomotor CD (using the blanking task) in 49 participants with schizophrenia or schizoaffective disorder (SZ), 36 bipolar participants with psychosis (BPP), and 40 healthy controls (HC). Participants made a saccade to a visual target. Upon saccade initiation, the target disappeared and reappeared at a horizontally displaced position. Participants indicated the direction of displacement. With intact CD, participants can make accurate perceptual judgements. Otherwise, participants may use saccade landing site as a proxy of pre-saccadic target to inform perception. Thus, multi-level modeling was used to examine the influence of target displacement and saccade landing site on displacement judgements.

**Study Results:**

SZ and BPP were equally less sensitive to target displacement than HC. Moreover, regardless of diagnosis, SZ and BPP with more severe positive symptoms were more likely to rely on saccade landing site.

**Conclusions:**

These results suggest that altered CD may be a trans-diagnostic mechanism of psychosis.

## Introduction

A basic sense of self—the implicit awareness of the “mineness” of experience—has been described as a fundamental process that underpins subjective experience.^1^ Perceived agency—the experience that I am in control of my actions—is central to the sense of self.^2^ Alterations in self and agency have been described as core features of schizophrenia spectrum disorders^3^ from which frank psychotic symptoms may emerge (eg, delusions of control).^4^

A basic sense of agency over movements relies on an internal model of one’s actions, a model that can be constructed using corollary discharge signals (CD). CD are “copies” of motor commands that are sent to sensory brain regions and used to compute predictions of imminent sensory consequences of that action.^4^ A mismatch between predicted and actual sensory inputs may lead to the (erroneous) inference that the sensation was caused by external forces (rather than one’s own actions). Altered CD has been posited as a basic mechanism of agency disturbances in schizophrenia,^4,5^ and this idea is supported by empirical findings across different sensory modalities.^6–9^

In the current study, we investigated whether altered CD signaling is specific to schizophrenia or may be a more general mechanism of psychosis. Importantly, there is increasing phenomenological, cognitive, and genetic evidence for a transdiagnostic psychosis spectrum.^10,11^ Indeed, ~50% of people with bipolar disorder experience psychosis.^12,13^ Thus, bipolar disorder patients with a history of psychosis might also have alterations in CD, though possibly to a lesser extent than people with schizophrenia. On the other hand, self-disturbances have traditionally been considered a pathognomonic feature of schizophrenia^14,15^—although this is contentious.^16^ Should agency disturbances be specific to the schizophrenia spectrum, we may expect altered CD in individuals with schizophrenia but not in those with bipolar disorder. To our knowledge, only one study has examined CD in bipolar participants with a history of psychosis, and found that the influence of CD in the auditory system was equivalently reduced in individuals with bipolar disorder and schizophrenia.^17^

Here, we investigated CD in the visuomotor system, as we believe it holds advantages over other sensory and motor systems. First, neurophysiology studies have outlined a specific circuit involved in generating and relaying CD signals associated with saccadic eye movements in non-human primates,^18^ and this work can potentially be leveraged to understand neurobiological mechanisms of agency. Second, robust psychophysics paradigms allow us to quantify the influence of CD signaling on visual perception.^19^ Individuals with schizophrenia show evidence for a reduced influence of CD in the visuomotor domain; these effects have been related, albeit inconsistently, to positive symptom severity.^7,19^

**Fig. 1.**
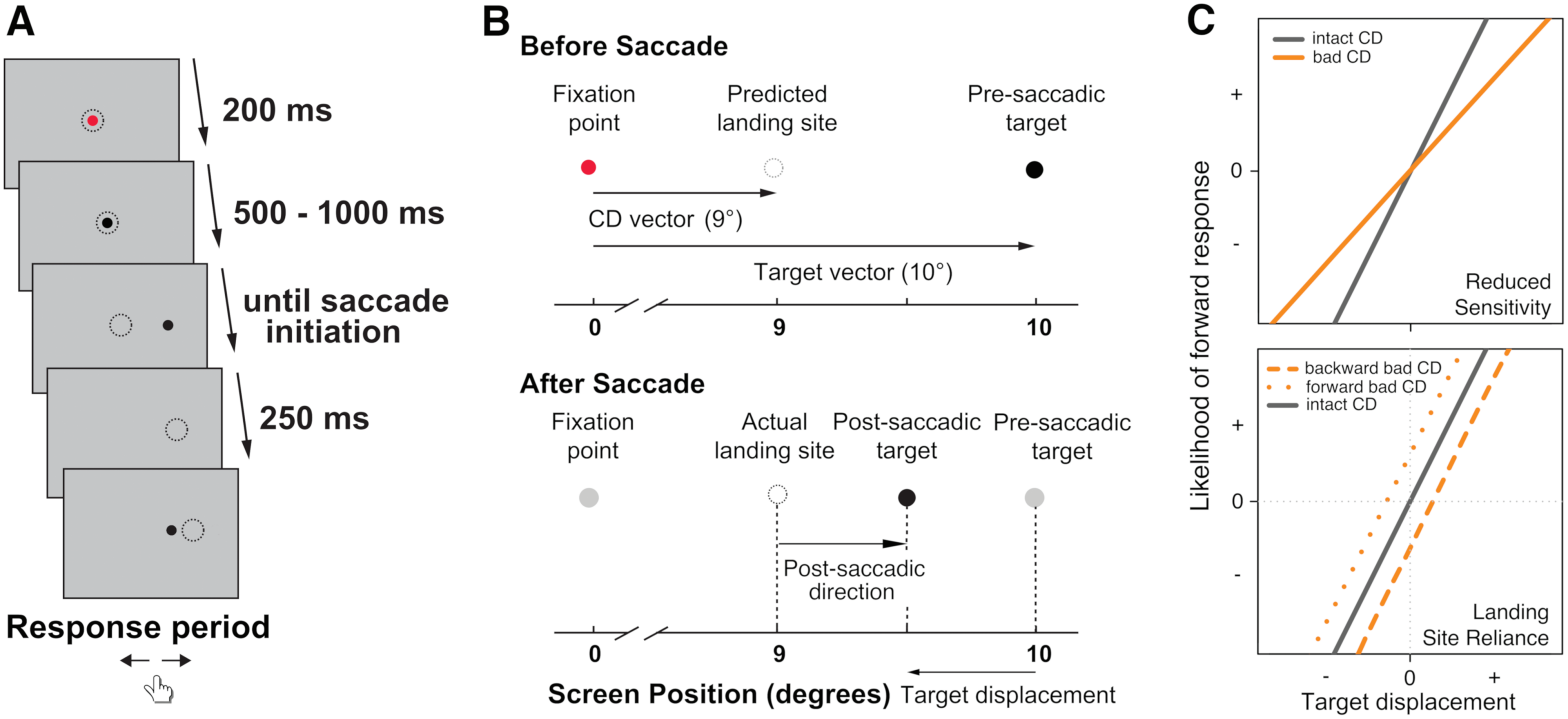
(A) Task schematics. Dotted circles represent gaze positions and do not appear on the screen. (B) Using corollary discharge (CD) signals to inform perceptual judgments. Top, when participants initiate a saccade, they also compute a CD vector containing saccade information; participants can use CD to predict that the saccade landing site will fall short of the target. Bottom, after the target disappears and reappears at a displaced location, participants use CD to remap the pre-saccadic target location to judge the displacement direction (correct response here is backward). However, if CD is altered, participants may use saccade landing site as a proxy of the pre-saccadic target location, thereby erroneously reporting a forward displacement. Adapted from Collins et al., 2009. (C) Hypothesized regression results. Top, less reliance on CD may lead to reduced sensitivity to target displacement (i.e., a flatter slope). Bottom, less reliance on CD may lead to more reliance on saccade landing site. This will be reflected in a positive intercept when the post-saccadic target location was forward to saccade landing site (i.e., a forward response bias), and/or a negative intercept when the post-saccadic target location was backward (i.e., a backward response bias)..

We used the blanking task, an established psychophysical paradigm, to assess oculomotor CD (figure 1A).^20^ In this task, a visual saccade target briefly disappears while a saccade is being executed and, briefly after, reappears at a displaced location. Participants judge the direction of the displacement relative to the original (pre-saccadic) location. Because saccades are often inaccurate, falling long or short of targets, *where* gaze lands (ie, saccade landing site) is not a reliable proxy of pre-saccadic location. To make a correct judgment of displacement direction, the system needs to remap the precise location of the pre-saccadic stimulus relative to gaze location. This remapping requires access to information about saccade kinematics that is conveyed by CD.^21^ Without such information, participants may use the saccade landing site as a proxy for stimulus location (ie, assume the eyes landed on the pre-saccadic target). Consistent with using CD to accurately inform perception, healthy participants make accurate perceptual judgements independent of saccade landing site.^21^ Disruptions in a neural pathway relaying oculomotor CD leads to a reliance on saccade landing site instead.^22,23^ Similarly, previous studies have found such a reliance in schizophrenia participants with more severe positive symptoms.^24,25^

We compared performance on the blanking task among 3 groups: (1) participants with schizophrenia or schizoaffective disorder, (2) bipolar participants with psychotic features, and (3) healthy control (HC) participants. We also examined the relationship between measures of CD and both clinical symptoms and anomalous self-experiences (ASE), which are present in the general population.^26,27^ We first aimed to replicate and expand findings of a reduced influence of CD in the blanking task in individuals with a schizophrenia spectrum disorder using more sophisticated statistical modeling.^24^ Given evidence from the auditory domain,^17^ we also hypothesized that participants with schizophrenia and bipolar with psychotic features would exhibit an increased reliance on saccade landing site relative to HC, and that this would be related to positive symptom severity. Lastly, we hypothesized that reliance on saccade landing site would be associated with ASE across groups.

## Methods

### Participants

Fifty-six participants with schizophrenia or schizoaffective disorder (SZ), 38 participants with bipolar disorder with psychotic features (BPP), and 40 HC completed the blanking task. Participants were recruited from outpatient mental health facilities, existing research registries and subject pools, and community advertisements. Diagnoses were based on an electronic version of the Structured Clinical Interview for DSM-5 (SCID-5)^28,29^ conducted by trained personnel, incorporating information from medical records and collateral informants, when available or deemed necessary. Final diagnosis was determined at a consensus meeting. Exclusion criteria for all participants included a history of head injury with loss of consciousness >1 h, neurological disorder, moderate or severe substance use disorder within the past 6 months, and vision that was not (corrected to) normal. HC were additionally excluded for personal history of mental illness or psychotropic use, and first-degree relatives with a history of schizophrenia spectrum or bipolar disorder. We excluded 9 participants due to poor task performance (see supplementary methods), resulting in a final sample of 49 SZ, 36 BPP, and 40 HC (Table 1). All participants gave written informed consent and were reimbursed for participation. The study was approved by the Michigan State University Institutional Review Board.

**Table 1.** Demographic and clinical information.

	SZ (*N* = 49)	BPP (*N* = 36)	HC (*N* = 40)		
	Mean (SD)	Mean (SD)	Mean (SD)	Statistics	*P*
Age (years)	34.9 (11.3)	36.3 (11.3)	35.5 (10.1)	*F* = 0.17	.85
Sex (female/male)	19/30	18/18	16/24	*χ* ^2^ = 1.21	.55
Race: Asian/Indian	0	1	5	*χ* ^2^ = 21.53	.018
Black	17	3	5		
Native American	0	1	0		
White	27	28	27		
Multiracial	3	1	2		
Other	2	2	1		
WTAR	100.3 (11.4)	106.7 (9.6)	110.6 (6.0)	*F* = 12.78	<.001
Education (years)	13.5 (2.0)	14.9 (2.2)	17.4 (2.9)	*F* = 28.64	<.001
Parental education (years)	14.9 (3.1)	16.0 (3.0)	15.4 (3.4)	*F* = 1.22	.30
IPASE	141.7 (45.5)	112.7 (40.0)	76.4 (20.7)	*F* = 24.88	<.001
Illness duration (Years)	10.6 (9.9)	12.9 (10.0)	—	*t* = 1.03	.31
Antipsychotics (Yes/No)	46/3	27/9	—	*χ* ^2^ = 6.10	.01
CPZ equivalent (mg)^62–64^	372.1 (393.9)	145.6 (152.1)	—	*t* = −3.56	.001
BPRS	46.0 (14.5)	35.3 (8.9)	—	*t* = −4.00	<.001
YMRS	9.8 (7.6)	6.2 (8.2)	—	*t* = −1.97	.053
HRSD	11.8 (8.1)	8.7 (6.3)	—	*t* = −1.73	.09
SAPS: Total score^a^	17.4 (16.6)	6.2 (8.5)	—	*t* = −3.93	<.001
Global summary^a^	5.8 (4.0)	2.8 (3.4)	—	*t* = −3.34	.001
Delusion^b^	7.1 (7.6)	1.9 (3.3)	—	*t* = −4.10	<.001
Hallucination^b^	5.6 (7.0)	1.1 (2.4)	—	*t* = −4.08	<.001
SANS: Total score	19.7 (16.4)	9.7 (9.6)	—	*t* = −3.41	.001
Global summary	6.9 (5.0)	4.2 (3.8)	—	*t* = −2.62	.011
SAPP lifetime	2.7 (2.8)	1.8 (2.5)	—	*t* = −1.43	.16
current	1.0 (2.2)	0.1 (0.4)	—	*t* = −2.63	.011

*Notes*: BPP, participants with bipolar disorder with psychotic features; BPRS, Brief Psychiatric Rating Scale; CPZ, chlorpromazine; HC, healthy controls; HRSD, Hamilton Rating Scale for Depression; IPASE, Inventory of Psychotic-like Anomalous Self-Experiences; SANS, Scale for the Assessment of Negative Symptoms; SAPP, Scale for the Assessment of Passivity Phenomena; SAPS, Scale for the Assessment of Positive Symptoms; SZ, participants with schizophrenia or schizoaffective disorder; WTAR, Wechsler Test for Adult Reading; YMRS, Young Mania Rating Scale.

^a^Total score: sum of individual symptom ratings; Global summary: sum of global item ratings.

^b^Sum of items loading on factors related to delusions (ie, bizarre delusions, other delusions, and paranoid delusions) and hallucinations (ie, non-auditory hallucinations, and auditory hallucinations).^35^

### Assessments

We assessed clinical symptoms using the Scale for the Assessment of Positive Symptoms (SAPS),^30^ Scale for the Assessment of Negative Symptoms (SANS),^31^ Young Mania Rating Scale (YMRS),^32^ Hamilton Rating Scale for Depression (HRSD),^33^ and Brief Psychiatric Rating Scale (BPRS).^34^ We were particularly interested in the relationship between performance and positive symptoms. To examine positive symptoms in greater detail, separate hallucination and delusion scores were derived based on a factor analytic study of the SAPS and summing items loading on factors related to delusions (ie, bizarre delusions, other delusions, and paranoid delusions) and hallucinations (ie, non-auditory hallucinations, and auditory hallucinations).^35^ We measured premorbid IQ using the Wechsler Test for Adult Reading (WTAR).^36^ To assess agency-related phenomena, we used the Scale for the Assessment of Passivity Phenomena (SAPP)^37^ in SZ and BPP and the Inventory of Psychotic-like Anomalous Self-Experiences (IPASE)^38^ in all participants. The SAPP is an interview-based measure of current and lifetime passivity experiences,^1^ and the IPASE is a self-report measure of subjective self-disturbances. See supplementary methods for information on missing data.

## Blanking Task

### Apparatus and Setup

Participants sat in a dimly lit room with their head stabilized 59 cm in front of a computer screen (screen size: 393 × 292 mm; spatial resolution: 1280 × 960 pixels; refresh rate: 85 Hz). The only sources of light in the room were the infrared light from the eye-tracker and the computer screen with its brightness set at the lowest. A pillow was set by the door to block off external lights. We used an EyeLink 1000 to track eye position (SR Research, Ottawa, Ontario, Canada). Participants used a computer keyboard to respond. We used Psychophysics^39^ and EyeLink^40^ toolboxes via MATLAB (MathWorks, Portola Valley, CA) to present the stimulus and collect responses.

### Design and Procedure

This task measures the degree to which CD influences visual perception. Each trial started with participants fixating on a red dot with a diameter of 0.2° visual angle presented on a gray background (figure 1A). To reduce anticipation effects and stereotypical behavior,^21^ the dot appeared randomly with equal probability at 1 of 9 fixation locations (–1°, 0°, or 1° displacement horizontally and vertically relative to the center of the screen). Once participants maintained fixation for 200 ms, the dot turned black and stayed on for another random period between 500 and 1000 ms. Then it disappeared and reappeared 10° to the left or right of the fixation location (pre-saccadic location). Participants were instructed to look at the target as quickly as possible. Once they initiated a saccade, the dot disappeared for 250 ms (ie, blanked) and reappeared at a new location (post-saccadic location). The post-saccadic location was 0.25°, 0.50°, 1.00°, 1.50°, 2.00°, or 3.00° of visual angle to the left or right of the pre-saccadic location, or in the exact same location (ie, 0° displacement). The closest distance between a post-saccadic location and the screen border was 7.17° visual angle. The dot then stayed on the screen until participants responded via a key press indicating the direction the dot jumped relative to the pre-saccadic location (left or right). For analysis, responses were recoded as forward when the target was perceived to jump further from fixation and backward when perceived as jumping closer to fixation. Therefore, the outcome variable of this task was dichotomous (ie, participants’ judgment of target jumping forward or backward). There were a total of 234 trials (9 fixation locations × 2 saccade directions × 13 post-saccadic displacements) per participant. The intertrial interval was self-paced and the task typically took 30 min to complete. See supplementary methods for saccade detection and performance exclusion criteria.

### Statistical Analysis

We used 1-way ANOVAs to compare groups on age, IQ, years of education (self and parental), and IPASE, and *χ*2 tests to compare groups on sex and race. We used independent *t* tests to compare SZ and BPP participants on clinical symptoms, CPZ equivalent dosages, and years of illness, and *χ*2 tests on proportion of participants currently using antipsychotics. We used SPSS Statistics version 27.0 (IBM, Armonk, NY) for these analyses.

We constructed multilevel binary logistic models with maximum pseudo-likelihood to predict participants’ responses on each trial (forward/backward). Statistical analyses are described in more detail in supplementary methods. We defined 2 variables to characterize saccade landing site: post-saccadic direction and post-saccadic distance (figure 1B). Post-saccadic direction describes the direction of post-saccadic target location relative to saccade landing site (forward: post-saccadic target further away from fixation than saccade landing site; backward: post-saccadic target closer to fixation than saccade landing site). If participants relied on saccade landing site to approximate pre-saccadic location, we hypothesized a high likelihood of forward responses on trials with a forward post-saccadic direction and backward responses on trials where the target appeared backward of the saccade landing site. Post-saccadic distance describes the distance between post-saccadic location and saccade landing site (regardless of the direction).

In all models, we included target displacement as a predictor and coded forward jumps as positive and backward jumps as negative. Therefore, we expected that the proportion of forward responses would increase as target displacement increased. We also included post-saccadic direction and post-saccadic distance to examine participants’ potential reliance on saccade landing site (instead of CD information) when making perceptual judgments.

The regression parameter that most directly reflects CD functioning is post-saccadic direction: a significant effect or interaction involving post-saccadic direction would indicate that participants relied on saccade landing site (instead of CD information) when making perceptual judgments. More specifically, this reliance would be reflected in the intercept when examining forward and backward landing trials separately. The intercept reflects participants’ perceived target displacement when the actual displacement is 0 (ie, when the target does not move). Therefore, this intercept parameter is conceptually equivalent to the psychophysical parameter of perceptual null location. A positive intercept suggests a bias toward forward responses, and a negative intercept suggests a bias toward backward responses. The other parameter that likely relates to CD functioning is the slope of target displacement. We expected a strong relationship between target displacement and perceptual judgements (ie, the larger the displacement forward, the more likely participants would report a forward jump). This slope parameter is conceptually equivalent to the psychophysical parameter of just noticeable difference. In other words, increased values of slope reflect greater perceptual sensitivity. While the slope may capture other basic visual processing components, a reliance on saccade landing site is expected to result in less reliance on target displacement, and thus a flatter slope.

In the first model, we examined group differences in blanking task performance. We included all main effects and interactions among group, target displacement, post-saccadic direction, and post-saccadic distance. We hypothesized a reduced influence of CD signals on trans-saccadic perception in SZ and BPP, leading to (1) an attenuated target displacement effect (ie, a flatter slope; figure 1C, top) due to a decreased reliance on target displacement; and (2) an increased reliance of perceptual judgements on saccade landing site. In other words, in SZ and BPP, we predicted an increased likelihood of forward responses on trials where the post-saccadic target appeared forward of participants’ gaze location (ie, a positive intercept), and a decreased likelihood of forward responses on backward trials (ie, a negative intercept; figure 1C, bottom). As CD signaling also affects corrective saccades, we also examined corrective saccades to the pre-saccadic target and whether they differed between groups in exploratory analyses (see supplementary results).

Next, we examined the effect of clinical symptom severity on task performance in SZ and BPP only. We preserved the group variable in all symptom models to examine variance explained by symptoms over and above that explained by diagnosis. Specifically, we examined the effects of SAPS total score in a primary analysis, given our hypothesis that altered CD may underly positive symptoms. The effect of SANS total score was examined to assess symptom specificity. Lastly, we examined the effect of ASE on task performance across the entire sample given the posited relationship between altered CD and ASE. For each model, we included the score as both a main effect and a moderator of the effects of task factors. We hypothesized that participants with more severe positive symptoms and ASE would exhibit task performance consistent with a decreased influence of CD on visual perception.

In all multilevel models, we coded participant response as 1 = forward and 0 = backward. Post-saccadic distance was grand mean centered. Post-saccadic direction was effect coded as −1 = forward and 1 = backward, and group as −1 = SZ, 0 = HC, and 1 = BPP. All symptom measures were grand mean centered. To account for individual differences in the effects of various task factors and general performance, we included the following random effects in all models except for the IPASE model: variances for the intercepts, and variances of the slopes for target displacement, post-saccadic direction, post-saccadic distance, saccade direction, target displacement × saccade direction, post-saccadic direction × saccade direction, and post-saccadic distance × saccade direction. For the IPASE model, we did not include variances of the slope for target displacement × saccade direction in the random effects due to too little variance leading to the model not converging. We included saccade direction in random effects due to significant individual variability in its effect on task performance. We effect coded the variable as −1 = leftward and 1 = rightward. To follow up on significant interactions, we computed simple slopes for separate conditions.^41^ For easier interpretation of the parameters in log odds, we reported corresponding odds for all analysis results. We used SAS version 9.4 (SAS Institute, Cary, NC) for all multilevel modeling.

## Results

### Participant Characteristics and Saccade Metrics

Groups did not differ on age, sex, and parental education, but differed significantly on IPASE total score (SZ > BPP > HC). BPP and SZ did not differ on illness duration. Compared with BPP, SZ had more severe symptoms, higher rates of antipsychotic use, and higher CPZ equivalent doses. Participants did not differ on basic saccade kinematics or number of usable trials (Table 2).

**Table 2.** Saccade metrics and task performance.

	SZ (*N* = 49)	BPP (*N* = 36)	HC (*N* = 40)	*F*	*P*
	Mean (SD)	Mean (SD)	Mean (SD)		
Mean saccade amplitude (°)	9.02 (0.85)	9.18 (0.95)	9.27 (0.73)	1.02	.36
Mean reaction time to first saccade (ms)	231.53 (72.30)	247.10 (125.41)	233.26 (66.63)	0.36	.70
Mean saccade duration (ms)	54.88 (10.80)	55.03 (18.16)	49.84 (5.52)	2.36	.10
Percentage of invalid trials	4.29% (5.17%)	3.29% (4.85%)	2.39% (3.73%)	1.84	.16

*Notes*: BPP, participants with bipolar disorder with psychotic features; HC, healthy controls; SZ, participants with schizophrenia or schizoaffective disorder.

### Group Differences in Blanking Task

Full results from all multilevel models are presented in supplementary tables S2–S6. We found significant main effects of target displacement, *P* < .0001; group, *P* = 0007; post-saccadic direction, *P* < .0001; and post-saccadic distance, *P* < .0001 on perceptual judgments. As target displacement increased, the odds of making a forward response also increased when holding other predictors constant across groups. Next, we computed simple slopes for each group separately (see supplementary table S7 for full model results), and found that when target displacement was 0°, HC and BPP were significantly more likely to make backward responses (odds of forward response: HC = 0.44, *P* = .0002; BPP = 0.45, *P* = .0003), whereas SZ had no response bias (*P* = .80). In other words, HC and BPP, but not SZ, had a bias to report backward displacement (see supplementary discussion for interpretations). We then computed simple slopes for post-saccadic locations that were forward and backward of saccade landing sites separately. Participants were more likely to make a backward response when the post-saccadic target fell backward (compared with forward) to the saccade landing site. Lastly, the effect of post-saccadic distance can be better understood in the context of the target displacement × post-saccadic distance interaction effect (detailed in supplementary results).

**Fig. 2.**
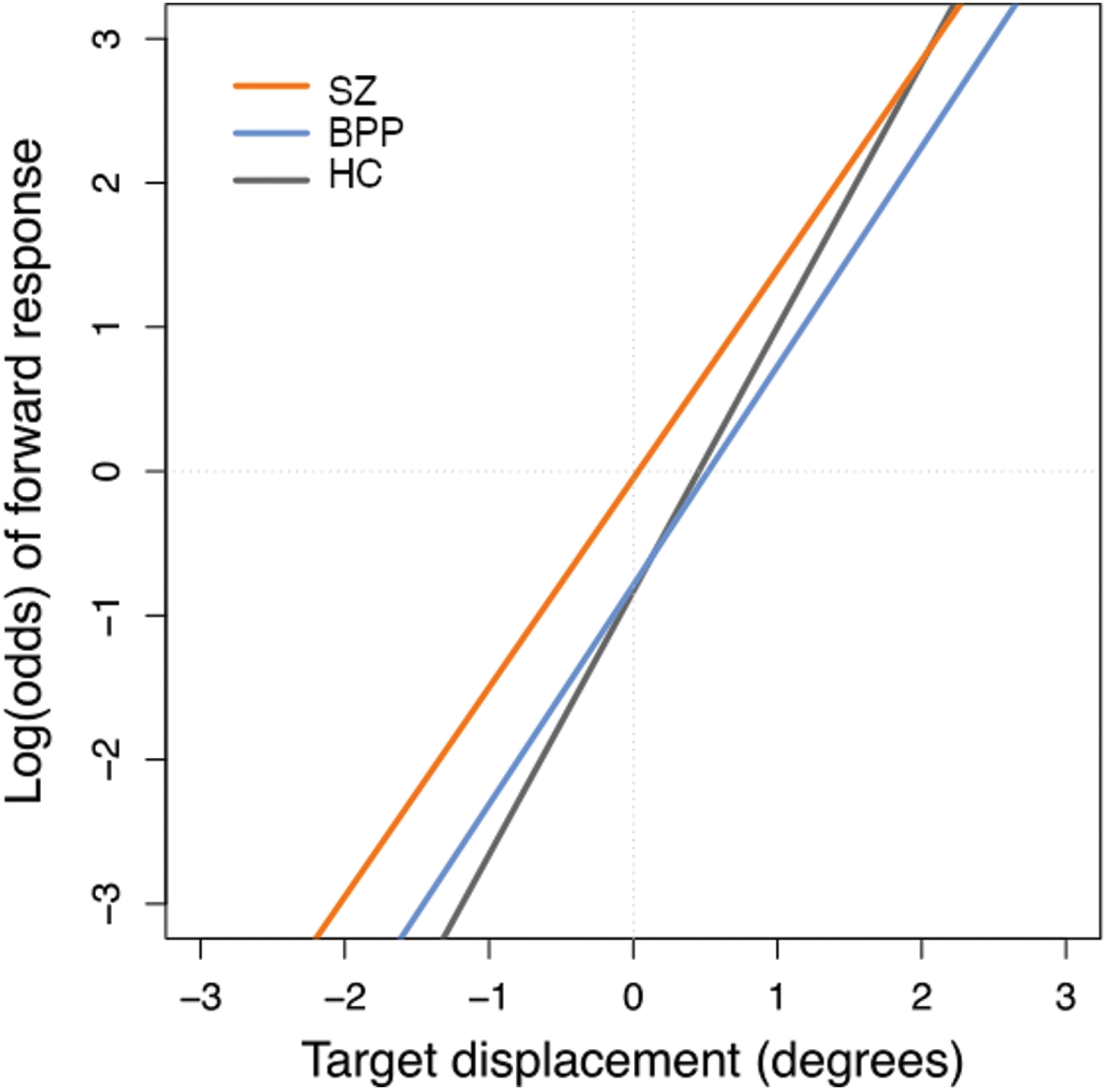
Group × target displacement interaction. The vertical axis indicates the likelihood of making a forward response.

Finally, there was a significant group × target displacement effect, *P* = .02. The effect of target displacement was statistically significant for all groups, but stronger in HC (odds = 6.35, *P* < .0001) than in BPP (odds = 4.69, *P* < .0001) and SZ (odds = 4.48, *P* < .0001). In other words, HC had higher sensitivity to target displacement than SZ and BPP (figure 2).

### Moderation Effects of Clinical Symptoms

**Fig. 3.**
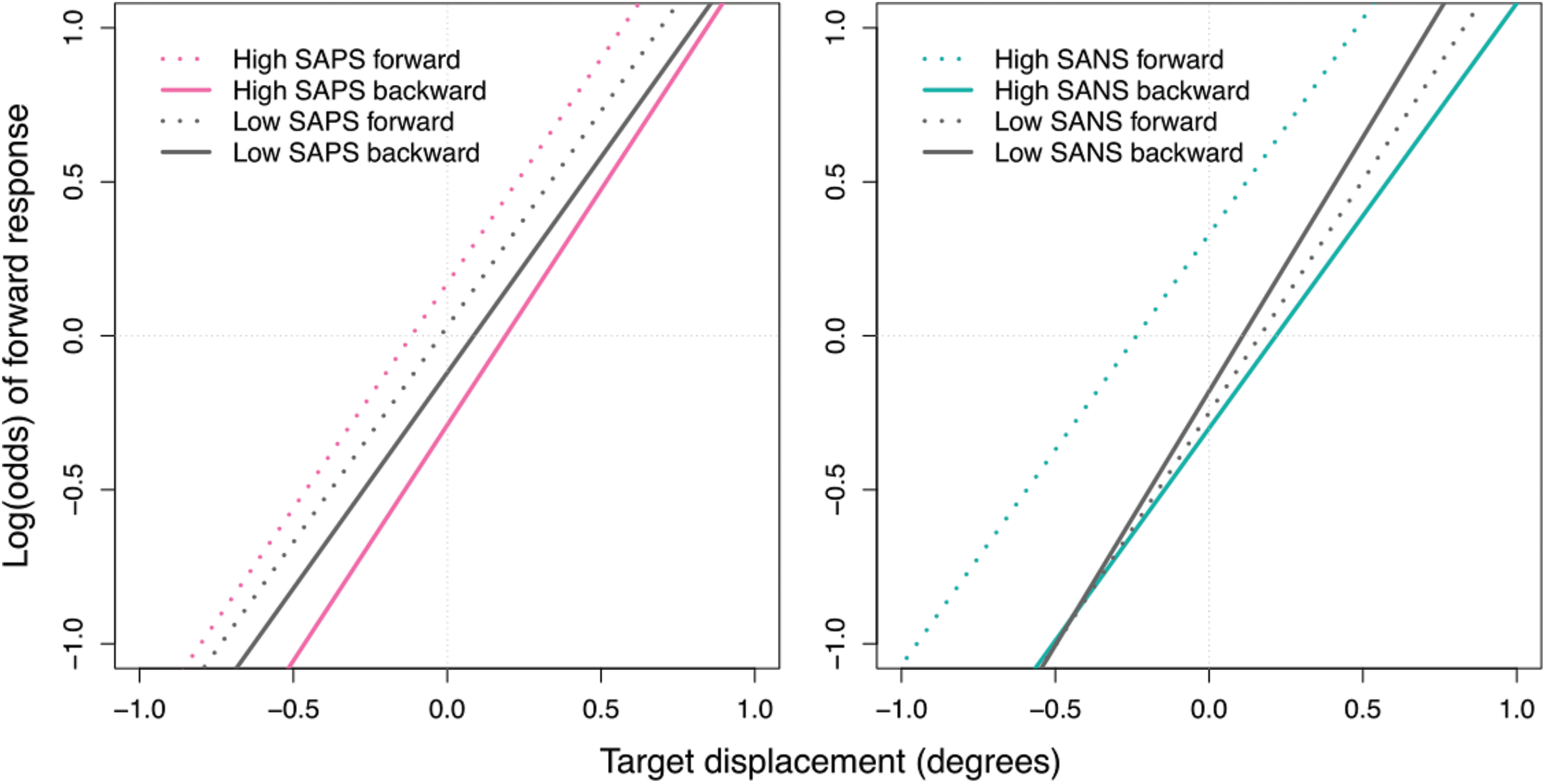
(A) Positive symptoms × post-saccadic direction interaction. (B) Negative symptoms × target displacement × post-saccadic direction interaction. Forward and backward refer to post-saccadic direction.

First, we examined the effect of positive symptoms (SAPS) on task performance in SZ and BPP only. We identified a significant SAPS × post-saccadic direction interaction, *P* = .02. To explore this interaction, we calculated estimates based on high and low SAPS score.^2^ The moderation effect of post-saccadic direction was only significant in participants with high SAPS scores, *P* < .0001 (low SAPS: *P* = .11). To better understand the effect of post-saccadic direction in participants with high SAPS scores, we computed simple slopes for post-saccadic locations that were forward and backward of saccade landing sites separately. We found that participants were more likely to make a backward response when the post-saccadic target fell backward (compared with forward) to the saccade landing site (see figure 3A). In other words, participants with more severe positive symptoms were more likely to rely on saccade landing site (instead of CD information) when making perceptual judgments. See supplementary results for post-hoc exploratory analyses of the effects of delusions and hallucinations on task performance, separately.

Next, we examined the effect of negative symptoms (SANS) on task performance in SZ and BPP only. We identified a significant SANS × target displacement × post-saccadic direction interaction, *P* = .04. To explore this interaction, we computed simple slopes for post-saccadic locations that were forward and backward of saccade landing sites separately. The moderation effect of SANS × target displacement was only significant when the post-saccadic target fell backward to the saccade landing site, *P* = .03 (forward: *P* = .68). We then calculated estimates based on high and low (1 SD above and below the mean) SANS score on these backward trials. We found a significant target displacement effect for both high and low SANS groups, *P* < .0001, but with each 1° increase in target displacement, the odds of participants making a forward response was higher when SANS was low vs high (4.21 vs 2.97). In other words, when the post-saccadic target fell backward to the saccade landing site, participants with less severe negative symptoms were more sensitive to target displacement than those with more severe symptoms (see figure 3B).

Lastly, we examined the effect of ASE (IPASE) on task performance using the entire sample. We did not identify any significant interaction effects involving ASE. See supplementary results for post-hoc exploratory analysis of effects of agency-related IPASE items on task performance.

## Discussion

In this study, we measured the ability to remap visual targets following an eye movement—a function that critically relies on intact CD signaling—in SZ, BPP, and HC. Results replicate previous findings in SZ and shed light on the transdiagnostic nature of CD impairments. We found that SZ and BPP had a similarly reduced influence of CD on visual perception that was trans-diagnostically associated with more severe positive symptoms. This finding is consistent with the notion that CD abnormalities may be a low-level mechanism of psychosis. In the following section, we discuss these findings, their implications, and limitations.

We predicted that a reduced influence of CD signals would impair the ability to accurately remap a visual stimulus following a saccadic eye movement, and thus lead to a *greater* reliance on saccade landing site (rather than a remapped representation of the pre-saccadic target) to inform perceptual judgements about the direction of a post-saccadic target displacement. Consequently, we predicted that impaired CD signaling would also lead to attenuated sensitivity to the post-saccadic target jump due to a *decreased* reliance on target displacement. Using a larger sample and statistical modeling informed by trial-level data, our findings replicate those from previous studies^24,25^ showing that SZ have reduced sensitivity to target displacement, therefore suggesting a reduced influence of CD signals on visual perception. BPP had an equivalent degree of reduction in sensitivity as SZ, consistent with the previously reported reduced influence of CD in the auditory domain in BPP.^17^ Although the degree to which participants relied on saccade landing site did not differ across groups, it did depend on symptom severity. Participants with more severe positive symptoms were more likely to rely on saccade landing site when making perceptual judgments, which replicates and expands upon previous findings in SZ.^24,25^ Critically, the effect of positive symptom severity was not explained by diagnosis.

The association between positive symptoms and CD alterations may be explained by the broader role of CD in supporting a sense of agency.^4^ That is, positive symptoms and alterations in trans-saccadic perception may share a common cause. An alternative explanation is that the association between oculomotor CD and positive symptoms may be mediated by altered visual stability. CD plays a critical role in maintaining the perception of a stable world despite eye movements causing displacement of retinal images.^42^ Consequently, oculomotor CD alterations may lead to an illusory perception of movements of objects or scenes, or even a perception of a fragmented and unstable world at large.^7^ Indeed, these are documented visual distortions in people with or at-risk for psychosis, which are part of a broader class of subtle, subjectively experienced disturbances that may engender full psychotic symptoms.^43^ In this way, oculomotor CD alterations may contribute to positive symptoms without a direct link with self-disturbances.

The finding that BPP had equally attenuated sensitivity to target displacement as SZ is consistent with a previous finding of equivalently reduced auditory CD in BPP and SZ.^17^ It remains to be seen whether altered CD in other sensory modalities in SZ are also affected to the same extent in BPP (eg, proprioception, somatosensation).^44,45^ Nonetheless, the current finding might contribute to the debate of whether self-disturbances are pathognomonic to schizophrenia. First-rank symptoms can occur in BPP too,^46^ and earlier studies revealed that they did not distinguish schizophrenia from other psychotic disorders.^47^ Yet recent studies found that basic self-disturbances could distinguish schizophrenia from BPP^48^ and other mental illnesses.^49,50^ One potential reason for conflicting findings is that “self” is a multi-faceted construct, and anomalies within different facets may be more or less specific to schizophrenia.^16^ Current findings would suggest that mechanisms underlying the basic sense of agency—one component of selfhood—might be similarly altered across both non-affective and affective psychotic disorders. The association with positive symptom severity across diagnostic categories further supports the notion of a psychosis spectrum,^11^ and altered CD being a transdiagnostic illness mechanism.

Given theories positing CD signaling as a mechanism underlying the sense of agency and recent findings that IPASE scores were associated with altered CD in the auditory domain in schizophrenia,^51^ we hypothesized that reduced influence of CD on perceptual judgment would be associated with ASE. However, we did not find a moderation effect of IPASE scores on the performance of the blanking task. In interpreting this null effect, one explanation is that impaired CD signaling is simply not a robust mediator of ASE. However, it is also worth considering the various factors that may contribute to IPASE scores and task performance and that may obscure the relationship between ASE and measures of CD in the current study. Specifically, high IPASE scores are related to a wide range of psychopathology in people with schizophrenia, including emotional distress, psychosis, mania, and disorganization.^52,53^ Moreover, performance on this task may reflect more than the integrity of CD signals.

In considering group differences in and clinical associations with attenuated sensitivity to target displacement, we must consider factors aside from a reduced influence of CD that would lead to this reduced sensitivity. That is, the observed findings might reflect group differences in or associations with processes that may or may not be related to CD signaling. One such consideration is that the effective duration of the blank may have been shorter in people with psychosis. This is important because people cannot perceive small target displacements during a saccade without the target blanking for a short period (ie, trans-saccadic suppression effect).^20^ Thus, a shorter effective blank duration would be expected to lead to poorer localization of the post-saccadic target. However, we do not think this is a likely explanation for our findings as saccade duration was not significantly different across groups; thus, the duration between saccade completion and onset of the post-saccadic target was equivalent. However, we must also consider that people with schizophrenia have been found to have a longer visual persistence effect^54^ (but see ref. ^[55]^), which may decrease the effective blank duration. Again, we do not expect prolonged visual persistence to explain the current findings, as persistence effects are retinotopic and would therefore be stronger during fixation. Regardless, given recent findings that indicate that non-CD related processes can explain the complete pattern of findings from trans- and peri-saccadic localization studies,^56,57^ the contribution of basic visual impairments to poorer task performance must be considered. Similarly, non-CD-related processes may account for the observed relationship between reduced sensitivity to target displacement and more severe negative symptoms in both SZ and BPP, which stand in contrast to previous studies in which we did not observe such a relationship.^24^ For example, factors like reduced motivation or more general visual impairments, which have been shown to correlate with negative symptoms in previous studies,^58^ may account for this relationship. However, one cannot completely rule out the possibility of altered CD being a shared mechanism of positive and negative symptoms. More research is needed to tease out whether this association between negative symptoms and reduced sensitivity to target displacement is mechanistic or secondary to other factors.

It is furthermore worth pointing out that the blanking task only assessed participants’ ability to use CD to make explicit perceptual judgements, but CD is also utilized for pre-attentive adjustments of actions. For example, it has been found that individuals with schizophrenia were able to adjust their motor actions despite being unable to verbally report a mismatch between predicted and actual sensory outcomes.^59^ But in another oculomotor paradigm, individuals with schizophrenia made slower and more infrequent corrective saccades to adjust for imprecise initial saccades.^60^ More studies are needed to investigate different functions that CD serves in action and perception, and how they may be differentially impacted in individuals with schizophrenia.

Several limitations need to be considered when interpreting the current findings. First, the current sample is a stable outpatient population that were experiencing only minimal passivity phenomena; more symptomatic samples may reveal stronger relationships between CD alterations and those symptoms that most clearly reflect alterations in agency, and better able to answer whether CD alterations are specifically related to agency disturbances. Second, most participants in this study were taking antipsychotics. Therefore, we cannot rule out potential confounding effects of medications. However, BPP exhibited an equivalent level of CD alterations as SZ, despite being on a significantly smaller dose of antipsychotics. Despite these limitations, we believe that there are potentially impactful directions for future work. For example, future studies might investigate whether shared behavioral indices of CD signaling abnormalities between BPP and SZ are underpinned by shared neurobiological mechanisms.^61^ If proven to be so, this would have implications for symptom-specific transdiagnostic treatment.

In conclusion, we found that compared with HC, SZ and BPP had equivalent alterations in oculomotor CD signaling. Moreover, those with more severe positive symptoms in both groups exhibited more reliance on perceptual rather than CD information, suggesting a transdiagnostic association between CD alterations and psychosis. Together, these findings suggest that CD alterations may be a transdiagnostic marker of psychosis.

## Supplementary Material

sbad180_suppl_Supplementary_Material

## Footnotes

1 We did not examine the effect of SAPP current total score because the majority of participants scored a 0. Although we found significant interaction effects involving SAPP lifetime score, the restricted range, and low SAPP score values limit the generalizability of the results. Therefore, the analysis and results involving SAPP is reported in supplementary methods and results.

2 We used 12 above and below the mean as high and low SAPS scores. We did not use 1 SD above and below the mean because 1 SD (15) is larger than the grand mean of SAPS (13). Therefore, we picked a value that is large enough to show the interaction effect but would not result in an invalid SAPS score.
