# Supplementary material for: Abnormal Oculomotor Corollary Discharge Signaling as a Trans-diagnostic Mechanism of Psychosis
Source: Schizophr Bull. 2024 Jan 20;50(3):631–41. doi: 10.1093/schbul/sbad180 (PMC11059795; doi:10.1093/schbul/sbad180)
Supplement: sbad180_suppl_Supplementary_Material [file sbad180_suppl_supplementary_material.docx]

**Supplementary Methods**

**Missing data**

Seven participants (2 SZ and 5 BPP) did not have SAPS and SANS data. Three participants (2 SZ and 1 BPP) did not have SAPP data. IPASE data were missing for 20 participants (10 SZ, 3 BPP, and 7 HC). Out of 57 items of IPASE, four participants missed one item, two participants missed two items, and one participant missed seven items. No single item missed data from more than two participants. Therefore, person-mean imputation was deemed the appropriate method to calculate a total score for these participants.^1–3^ We conducted the analysis with and without these seven participants. Results from the model including imputed scores were reported in the main text and Supplementary Table S6. Results excluding imputed scores were essentially the same.

**Blanking task**

**Saccade detection**

Online saccade detection followed a boundary technique: saccade initiation was defined as eyes leaving a 2° circular boundary around the fixation location. The same boundary was used to enforce stable fixation at the beginning of each trial until the initial target displacement. Participants received a warning message whenever they initiated a saccade before the initial target displacement, and then repeated the trial.

We identified all saccades offline using the automated EyeLink procedure (saccade detection velocity threshold: 30 degrees/sec, acceleration threshold: 8000 degrees/sec^2^, motion threshold: 0.1 degrees). Then we identified response saccades as the first saccade meeting the following criteria: 1) initiated more than 100 ms after onset of pre-saccadic target; 2) larger than 1° visual angle; 3) endpoint closer than 8° to the pre-saccadic location. We excluded trials with no identifiable response saccades.

**Performance exclusion criteria**

We excluded participants based on two indices suggesting inappropriate task performance. We fit a logistic function to each individual’s data describing proportion of forward response at each target displacement (across fixation positions and saccade directions; individual functions and group averages can be seen in Figure S1). First, we obtained the perceptual null location (PNL) per participant from these functions (the displacement where participants reported equal amount of forward and backward responses, indicating no difference between the pre-saccadic and post-saccadic target locations). We excluded all participants with an absolute value of PNL larger than 4 (i.e., reflecting a poor function fit). Second, we calculated the difference between y values (i.e., proportion of forward response) corresponding to the biggest and smallest displacement (i.e., 3° and -3°) for each participant using the functions. We excluded all participants with a difference smaller than 0.4 (i.e., reflecting very little rise in the function, suggestive of poor discrimination between forward and backward displacements). Using these two criteria, we excluded 9 participants (7 SZ and 2 BPP) in total.

**Statistical analysis**

For the SAPP model, we included SAPP lifetime total score as a main effect and a moderator of the effects of task factors. We hypothesized that SZ and BPP participants with more severe passivity symptoms would exhibit task performance consistent with a decreased influence of CD on visual perception (i.e., attenuated sensitivity to target displacement and/or a reliance on saccade landing site).

To follow up on the significant findings of the SAPS model, we examined the effect of delusion and hallucination severity on task performance in two separate models. The models are otherwise the same as the SAPS model except for the variable of clinical symptom severity (i.e., substituting SAPS total score with delusion and hallucination severity in each model).

Lastly, we conducted an exploratory analysis examining the effect of agency-related items of IPASE on task performance. We computed the sum of all items that loaded on the self-awareness and presence factor.^4^ Two participants missed one item and one participant missed four items from this subscale. We used person-mean imputation to calculate a subscale total score for these participants. We conducted the analysis with and without these three participants. The model is otherwise the same as the IPASE model except that we used the Self-Awareness and Presence subscale total instead of the IPASE total score.

**Supplementary Results**

**Corrective saccade analyses**

We defined a corrective saccade as the first saccade following the initial response saccade that brought the eye closer to the post-saccadic target location, with an onset earlier than 80 ms after the onset of post-saccadic target (so that it could not be influenced by the visual information of the post-saccadic target location). In total, 93 participants (74.4% of the whole sample) made at least one corrective saccade towards the pre-saccadic target (Table S1). On average, participants made a corrective saccade on 2.32% of the trials with a valid response, and the three groups did not differ in the percentage or amplitude of these corrective saccades. We also examined the threshold for executing a corrective saccade, determined by selecting all trials where a corrective saccade was initiated and identifying the minimum value of the landing site error (i.e., the absolute value of the distance between saccade landing site and the pre-saccadic target location). The three groups did not differ in threshold for corrective saccade initiation either. Lastly, we examined the response time of these corrective saccades after the offset of the initial response saccade and did not identify a group difference. Combined, these findings suggest that basic saccade kinematics of corrective saccades and the distance between gaze location and the pre-saccadic target that triggers a corrective saccade were similar among the three groups. Given the small number of corrective saccades (likely due to the short window within which the participant could make a corrective saccade before the post-saccadic target appeared), we hesitate to interpret these findings.

**Group differences in blanking task**

We found a significant target displacement × post-saccadic distance interaction effect, *F*(1,10222)=7.86, *p*=.005. We calculated estimates based on large (1 SD above the mean) and small (1 SD below the mean) post-saccadic distance. We found a significant target displacement effect for both large, *F*(1,374)=495.98, *p*<.0001, and small, *F*(1,197)=864.38, *p*<.0001, conditions, but with each 1° increase in target displacement, the odds of participants making a forward response was higher when the distance between the saccade landing site and post-saccadic target was small than when it was large (3.66 vs. 2.90). In other words, participants were more sensitive to target displacement when the post-saccadic locations appeared closer to the saccade landing site than when they appeared further away. The spatial uncertainty of the post-saccadic target increases with the post-saccadic distance (as it appears further away from the fovea). Although this spatial uncertainty is theoretically independent of CD functioning, higher uncertainty could increase task difficulty because participants need to compare the post-saccadic location with the remapped pre-saccadic location (supported by CD signaling). Indeed this is what we observed in typically developing children in a previous study using this task.^5^

**Moderation effect of lifetime passivity symptoms**

We examined the effect of lifetime passivity symptoms on task performance as measured by Scale for the Assessment of Passivity Phenomena (SAPP) in SZ and BPP participants only. We found a significant lifetime passivity symptoms x group x post-saccadic distance x post-saccadic direction interaction effect (Figure S2), *F*(1, 2648) = 5.05, *p* = .02. To explore this interaction, we calculated estimates based on high (2.3 above the mean) and low (2.3 below the mean) SAPP score.^[[1]](#footnote-1)^ We found that the moderation effect of group x post-saccadic distance x post-saccadic direction interaction was only significant in participants with low SAPP scores, *F*(1, 3184) = 6.08, *p* = .01 (high SAPP: *F*(1, 2130) = 0.25, *p* = .62). To better understand this interaction effect in participants with low SAPP scores, we then computed simple slopes for SZ and BPP groups separately. We found that the moderation effect of post-saccadic distance x post-saccadic direction interaction was only significant in BPP participants, *F*(1, 1542) = 7.12, *p* = .008 (SZ: *F*(1, 6383) = 0.91, *p* = .34). To better understand this interaction effect in BPP participants with low SAPP scores, we then calculated estimates based on large (1 SD above the mean) and small (1 SD below the mean) post-saccadic distance. We found that the moderation effect of post-saccadic direction was only significant when the post-saccadic distance was large, *F*(1, 830) = 10.00, *p* = .002 (small: *F*(1, 114) = 0.43, *p* = .51). Lastly, to better understand this moderation effect, we computed simple slopes for post-saccadic locations that were forward and backward of saccade landing sites separately. We found that participants were significantly more likely to make a backward response when the post-saccadic target fell backward to the saccade landing site, *F*(1, 515) = 13.81, *p* = .0002, but they were not more likely to make a positive response when the post-saccadic target fell forward, *F*(1, 98) = 2.52, *p* = .12. In sum, when lifetime passivity symptoms were more severe, the group differences in task performance were consistent across different task factor conditions; but when lifetime passivity symptoms were less severe, the task factors had different effects on task performance between SZ and BPP. Specifically, BPP exhibited a significant backward response bias when the post-saccadic target fell largely backward to the saccade landing site, while SZ did not exhibit any response bias regardless of post-saccadic distance or post-saccadic direction.

**Moderation effect of delusions**

We examined the effect of delusion severity on task performance in SZ and BPP. We identified a significant delusion × post-saccadic direction interaction, *p* = .019. To explore this interaction, we calculated estimates based on high and low delusion score.^[[2]](#footnote-2)^ The moderation effect of post-saccadic direction was only significant in participants with high delusion scores, *p* < .0001 (low delusion: *p* = .07). To better understand the effect of post-saccadic direction in participants with high delusion scores, we computed simple slopes for post-saccadic locations that were forward and backward of saccade landing sites separately. We found that participants were more likely to make a backward response when the post-saccadic target fell backward (compared with forward) to the saccade landing site (see Figure S3). In other words, participants with more severe delusions were more likely to rely on saccade landing site (instead of CD information) when making perceptual judgments.

**Moderation effect of hallucinations**

We examined the effect of hallucination severity on task performance in SZ and BPP. We did not identify any significant interaction effects involving hallucinations.

**Moderation effect of anomalous self-awareness and presence experiences**

We examined the effect of more agency related IPASE items (i.e., self-awareness and presence factor) on task performance in all participants. Results from the models including and excluding imputed scores are the same, and thus only the previous model will be reported here. We found a significant self-awareness and presence × target displacement interaction effect, *p* = .04. To explore this interaction, we calculated estimates based on high and low (1 SD above and below the mean) subscale scores. We found a significant target displacement effect for both high and low scoring groups, *p* < .0001, but with each 1° increase in target displacement, the odds of participants making a forward response was higher when the subscale score was low versus high (3.39 vs. 3.35). In other words, participants with less anomalous self-awareness and presence experiences were more sensitive to target displacement than those with more anomalous experiences.

**Supplementary Discussion**

Interestingly, HC and BPP exhibited a tendency to make backward judgment when target displacement was 0°, but SZ did not demonstrate such a response bias. Previous studies have identified a similar backward response bias (indexed by a positive perceptual null location or a negative intercept) in HC^6^ and typically developing children.^5^ This could potentially be explained by the representational momentum phenomenon in spatial memory: the judged final position of a moving target is usually slightly forward of the actual position in the direction of the anticipated (implied) motion, potentially due to our internalized understanding of the laws of physics.^7^ In the blanking task, the initial target jump from the fixation point to the pre-saccadic location could create a sense of implied motion, and participants thus may have a strong expectation for the final location of the moving target to be forward of their saccade landing site, leading to the representation of the pre-saccadic target being slightly forward to its actual location. Therefore, more post-saccadic targets would be perceived as jumping backward than forward, resulting in a slight backward response bias. Contrary to our previous study,^8^ we did not observe a backward response bias in SZ in this study. A lack of such bias may reflect a reduced influence from prior experience and knowledge (such as laws of physics in this case) and thus a more “veridical” perception of the visual world, not unlike the diminished susceptibility to the depth inversion illusion in SZ.^9^

**Table S1.** Corrective saccade metrics.

|  | SZ (*N* = 49) | BPP (*N* = 36) | HC (*N* = 40) |  |  |
| --- | --- | --- | --- | --- | --- |
|  | Mean (SD) | Mean (SD) | Mean (SD) | Statistics | *p* |
| % participants with corrective saccade(s) | 83.67% | 72.22% | 65% | *χ^2^* = 4.16 | .13 |
| % valid trials with a corrective saccade | 2.57% (3.61%) | 2.52% (3.58%) | 1.84% (4.60%) | *F* = 0.44 | .65 |
| Mean corrective saccade amplitude (°) | 2.14 (1.19) | 2.24 (1.21) | 2.19 (1.35) | *F* = 0.05 | .95 |
| Mean landing site error threshold (°) | 2.04 (1.48) | 2.21 (1.36) | 2.18 (1.61) | *F* = 0.12 | .89 |
| Mean RT after response saccade offset (ms) | 183.74 (70.96) | 185.70 (63.90) | 165.08 (74.73) | *F* = 0.72 | .49 |

**Table S2.** Logistic multilevel regression coefficients estimating effects of group, target displacement, post-saccadic direction, and post-saccadic distance on perceptual judgment on the blanking task.

| Variable | *b* | Odds | Standard Error | *F(df)* | *σ^2^* | *Wald Z* |
| --- | --- | --- | --- | --- | --- | --- |
| Fixed Effects |  |  |  |  |  |  |
| Intercept | -0.05 | 0.95 | 0.19 |  |  |  |
| Group |  |  |  | 5.09** (147) |  |  |
| Target Displacement | 1.45 | 4.26 | 0.09 | 821.59*** (176) |  |  |
| Post-saccadic Direction | -0.18 | 0.84 | 0.08 | 21.68*** (572) |  |  |
| Post-saccadic Distance | -0.11 | 0.90 | 0.05 | 15.25*** (873) |  |  |
| Group × Target Displacement |  |  |  | 4.13* (173) |  |  |
| Group × Post-saccadic Direction |  |  |  | 0.30 (501) |  |  |
| Group × Post-saccadic Distance |  |  |  | 0.52 (752) |  |  |
| Target Displacement × Post-saccadic Direction | 0.03 | 1.03 | 0.04 | 1.22 (4574) |  |  |
| Target Displacement × Post-saccadic Distance | -0.06 | 0.94 | 0.03 | 7.86** (10222) |  |  |
| Post-saccadic Direction × Post-saccadic Distance | -0.0004 | 0.9996 | 0.05 | 0.99 (6757) |  |  |
| Group × Target Displacement × Post-saccadic Direction |  |  |  | 1.72 (4369) |  |  |
| Group × Target Displacement × Post-saccadic Distance |  |  |  | 1.35 (9505) |  |  |
| Group × Post-saccadic Direction × Post-saccadic Distance |  |  |  | 0.89 (5620) |  |  |
| Post-saccadic Distance × Post-saccadic Direction × Target Displacement | -0.004 | 0.996 | 0.03 | 0.11 (12960) |  |  |
| Group × Target Displacement × Post-saccadic Distance × Post-saccadic Direction |  |  |  | 0.44 (12096) |  |  |
| Random Effects |  |  |  |  |  |  |
| Intercept |  |  |  |  | 1.46*** | 7.36 |
| Saccade Direction |  |  |  |  | 0.17*** | 5.76 |
| Target Displacement |  |  |  |  | 0.25*** | 6.19 |
| Post-saccadic Direction |  |  |  |  | 0.03* | 2.25 |
| Post-saccadic Distance |  |  |  |  | 0.01* | 1.92 |
| Saccade Direction × Target Displacement |  |  |  |  | 0.004 | 0.74 |
| Saccade Direction × Post-saccadic Direction |  |  |  |  | 0.04** | 2.76 |
| Saccade Direction × Post-saccadic Distance |  |  |  |  | 0.009* | 2.30 |

**p* < .05, ***p* < .01, *** *p* < .001.

**Table S3.** Logistic multilevel regression coefficients estimating effects of group, positive symptoms (measured by SAPS), target displacement, post-saccadic direction, and post-saccadic distance on perceptual judgment on the blanking task in schizophrenia and bipolar groups.

| Variable | *b* | Odds | Standard Error | *F(df)* | *σ^2^* | | *Wald Z* | |
| --- | --- | --- | --- | --- | --- | --- | --- | --- |
| Fixed Effects |  |  |  |  |  | |  | |
| Intercept | -0.05 | 0.95 | 0.20 |  |  | |  | |
| Group |  |  |  | 4.76* (84) |  | |  | |
| Target Displacement | 1.45 | 4.26 | 0.08 | 413.06*** (109) |  | |  | |
| Post-saccadic Direction | -0.15 | 0.86 | 0.08 | 17.42*** (243) |  | |  | |
| Post-saccadic Distance | -0.11 | 0.90 | 0.05 | 13.69*** (277) |  | |  | |
| SAPS | -0.0007 | 0.9993 | 0.01 | 0.05 (83) |  | |  | |
| Group × Target Displacement |  |  |  | 0.53 (109) |  | |  | |
| Group × Post-saccadic Direction |  |  |  | 2.74 (243) |  | |  | |
| Group × Post-saccadic Distance |  |  |  | 1.22 (277) |  | |  | |
| Group × SAPS |  |  |  | 0.03 (83) |  | |  | |
| Target Displacement × Post-saccadic Direction | 0.02 | 1.02 | 0.04 | 0.02 (2444) |  | |  | |
| Target Displacement × Post-saccadic Distance | -0.06 | 0.94 | 0.03 | 6.40* (5550) |  | |  | |
| Target Displacement × SAPS | 0.004 | 1.004 | 0.005 | 0.06 (111) |  | |  | |
| Post-saccadic Direction × Post-saccadic Distance | 0.01 | 1.01 | 0.05 | 0.91 (1458) |  | |  | |
| Post-saccadic Direction × SAPS | -0.006 | 0.994 | 0.005 | 5.84* (180) |  | |  | |
| Post-saccadic Distance × SAPS | -0.003 | 0.997 | 0.003 | 1.25 (221) |  | |  | |
| Group × Target Displacement × Post-saccadic Direction |  |  |  | 0.07 (2444) |  | |  | |
| Group × Target Displacement × Post-saccadic Distance |  |  |  | 0.10 (5550) |  | |  | |
| Group × Target Displacement × SAPS |  |  |  | 0.11 (111) |  | |  | |
| Group × Post-saccadic Direction × Post-saccadic Distance |  |  |  | 1.50 (1458) |  | |  | |
| Group × Post-saccadic Direction × SAPS |  |  |  | 1.35 (180) |  | |  | |
| Group × Post-saccadic Distance × SAPS |  |  |  | 0.01 (221) |  | |  | |
| Post-saccadic Distance × Post-saccadic Direction × Target Displacement | -0.008 | 0.992 | 0.03 | 0.04 (6099) |  | |  | |
| Target Displacement × Post-saccadic Direction × SAPS | 0.001 | 1.001 | 0.003 | 0.06 (2002) |  | |  | |
| Target Displacement × Post-saccadic Distance × SAPS | -0.001 | 0.999 | 0.002 | 0.09 (4006) |  | |  | |
| Post-saccadic Direction × Post-saccadic Distance × SAPS | -0.003 | 0.997 | 0.003 | 0.30 (1027) |  | |  | |
| Group × Target Displacement × Post-saccadic Distance × Post-saccadic Direction |  |  |  | 0.25 (6099) |  | |  | |
| Group × Target Displacement × Post-saccadic Direction × SAPS |  |  |  | 0.02 (2002) |  | |  | |
| Group × Target Displacement × Post-saccadic Distance × SAPS |  |  |  | 0.82 (4006) |  | |  | |
| Group × Post-saccadic Direction × Post-saccadic Distance × SAPS |  |  |  | 0.27 (1027) |  | |  | |
| Post-saccadic Distance × Post-saccadic Direction × Target Displacement × SAPS | -0.00003 | 0.99997 | 0.002 | 0.38 (4440) |  | |  | |
| Group × Target Displacement × Post-saccadic Distance × Post-saccadic Direction × SAPS |  |  |  | 0.40 (4440) |  | |  | |
| Random Effects |  |  |  |  |  | |  | |
| Intercept |  |  |  |  | | 1.51*** | | 5.85 |
| Saccade Direction |  |  |  |  | | 0.21*** | | 4.84 |
| Target Displacement |  |  |  |  | | 0.19*** | | 4.85 |
| Post-saccadic Direction |  |  |  |  | | 0.009 | | 0.69 |
| Post-saccadic Distance |  |  |  |  | | 0.01* | | 1.78 |
| Saccade Direction × Target Displacement |  |  |  |  | | 0.004 | | 0.49 |
| Saccade Direction × Post-saccadic Direction |  |  |  |  | | 0.04* | | 2.19 |
| Saccade Direction × Post-saccadic Distance |  |  |  |  | | 0.004 | | 1.07 |

**p* < .05, ***p* < .01, *** *p* < .001.

SAPS, Scale for the Assessment of Positive Symptoms.

**Table S4.** Logistic multilevel regression coefficients estimating effects of group, negative symptoms (measured by SANS), target displacement, post-saccadic direction, and post-saccadic distance on perceptual judgment on the blanking task in schizophrenia and bipolar groups.

| Variable | *b* | Odds | Standard Error | *F(df)* | | *σ^2^* | *Wald Z* |
| --- | --- | --- | --- | --- | --- | --- | --- |
| Fixed Effects |  |  |  |  | |  |  |
| Intercept | -0.10 | 0.90 | 0.20 |  | |  |  |
| Group |  |  |  | 5.50* (85) | |  |  |
| Target Displacement | 1.48 | 4.39 | 0.08 | 460.33*** (104) | |  |  |
| Post-saccadic Direction | -0.14 | 0.87 | 0.08 | 11.18** (223) | |  |  |
| Post-saccadic Distance | -0.11 | 0.90 | 0.05 | 16.61*** (266) | |  |  |
| SANS | 0.008 | 1.008 | 0.01 | 0.17 (92) | |  |  |
| Group × Target Displacement |  |  |  | 0 (104) | |  |  |
| Group × Post-saccadic Direction |  |  |  | 0.99 (223) | |  |  |
| Group × Post-saccadic Distance |  |  |  | 1.77 (266) | |  |  |
| Group × SANS |  |  |  | 0.95 (92) | |  |  |
| Target Displacement × Post-saccadic Direction | 0.03 | 1.03 | 0.05 | 0.19 (2599) | |  |  |
| Target Displacement × Post-saccadic Distance | -0.06 | 0.94 | 0.03 | 14.20*** (6807) | |  |  |
| Target Displacement × SANS | -0.006 | 0.994 | 0.005 | 2.79 (126) | |  |  |
| Post-saccadic Direction × Post-saccadic Distance | 0.03 | 1.03 | 0.05 | 0.71 (1953) | |  |  |
| Post-saccadic Direction × SANS | -0.01 | 0.99 | 0.005 | 2.76 (416) | |  |  |
| Post-saccadic Distance × SANS | -0.005 | 0.995 | 0.003 | 3.40 (498) | |  |  |
| Group × Target Displacement × Post-saccadic Direction |  |  |  | 1.52 (2599) | |  |  |
| Group × Target Displacement × Post-saccadic Distance |  |  |  | 1.02 (6807) | |  |  |
| Group × Target Displacement × SANS |  |  |  | 0.38 (126) | |  |  |
| Group × Post-saccadic Direction × Post-saccadic Distance |  |  |  | 2.59 (1953) | |  |  |
| Group × Post-saccadic Direction × SANS |  |  |  | 0.22 (416) | |  |  |
| Group × Post-saccadic Distance × SANS |  |  |  | 0.26 (498) | |  |  |
| Post-saccadic Distance × Post-saccadic Direction × Target Displacement | -0.01 | 0.99 | 0.03 | 0.04 (8217) | |  |  |
| Target Displacement × Post-saccadic Direction × SANS | -0.003 | 0.997 | 0.003 | 4.03* (6460) | |  |  |
| Target Displacement × Post-saccadic Distance × SANS | -0.0008 | 0.9992 | 0.002 | 0.25 (10020) | |  |  |
| Post-saccadic Direction × Post-saccadic Distance × SANS | -0.008 | 0.992 | 0.003 | 2.66 (3772) | |  |  |
| Group × Target Displacement × Post-saccadic Distance × Post-saccadic Direction |  |  |  | 0.06 (8217) | |  |  |
| Group × Target Displacement × Post-saccadic Direction × SANS |  |  |  | 1.65 (6460) | |  |  |
| Group × Target Displacement × Post-saccadic Distance × SANS |  |  |  | 0.02 (10020) | |  |  |
| Group × Post-saccadic Direction × Post-saccadic Distance × SANS |  |  |  | 0.58 (3772) | |  |  |
| Post-saccadic Distance × Post-saccadic Direction × Target Displacement × SANS | -0.0005 | 0.9995 | 0.002 | 0 (11537) | |  |  |
| Group × Target Displacement × Post-saccadic Distance × Post-saccadic Direction × SANS |  |  |  | 0.03 (11537) | |  |  |
| Random Effects |  |  |  |  | |  |  |
| Intercept |  |  |  |  | 1.48*** | | 5.83 |
| Saccade Direction |  |  |  |  | 0.20*** | | 4.79 |
| Target Displacement |  |  |  |  | 0.19*** | | 4.83 |
| Post-saccadic Direction |  |  |  |  | 0.02 | | 1.09 |
| Post-saccadic Distance |  |  |  |  | 0.009 | | 1.55 |
| Saccade Direction × Target Displacement |  |  |  |  | 0.003 | | 0.48 |
| Saccade Direction × Post-saccadic Direction |  |  |  |  | 0.04* | | 2.26 |
| Saccade Direction × Post-saccadic Distance |  |  |  |  | 0.004 | | 1.11 |

**p* < .05, ***p* < .01, *** *p* < .001.

SANS, Scale for the Assessment of Negative Symptoms.

**Table S5.** Logistic multilevel regression coefficients estimating effects of group, lifetime passivity symptoms (measured by SAPP), target displacement, post-saccadic direction, and post-saccadic distance on perceptual judgment on the blanking task in schizophrenia and bipolar groups.

| Variable | *b* | Odds | Standard Error | *F(df)* | *σ^2^* | | *Wald Z* |
| --- | --- | --- | --- | --- | --- | --- | --- |
| Fixed Effects |  |  |  |  |  | |  |
| Intercept | -0.12 | 0.89 | 0.19 |  |  | |  |
| Group |  |  |  | 8.18** (93) |  | |  |
| Target Displacement | 1.41 | 4.10 | 0.08 | 614.52*** (118) |  | |  |
| Post-saccadic Direction | -0.20 | 0.82 | 0.08 | 21.37*** (281) |  | |  |
| Post-saccadic Distance | -0.11 | 0.90 | 0.06 | 18.97*** (459) |  | |  |
| SAPP | 0.005 | 1.005 | 0.07 | 0.07 (94) |  | |  |
| Group × Target Displacement |  |  |  | 0.93 (118) |  | |  |
| Group × Post-saccadic Direction |  |  |  | 0.92 (281) |  | |  |
| Group × Post-saccadic Distance |  |  |  | 2.22 (459) |  | |  |
| Group × SAPP |  |  |  | 0.12 (94) |  | |  |
| Target Displacement × Post-saccadic Direction | 0.007 | 1.007 | 0.05 | 0.16 (1834) |  | |  |
| Target Displacement × Post-saccadic Distance | -0.07 | 0.93 | 0.03 | 20.22*** (8928) |  | |  |
| Target Displacement × SAPP | 0.04 | 1.04 | 0.03 | 2.69 (142) |  | |  |
| Post-saccadic Direction × Post-saccadic Distance | -0.003 | 0.997 | 0.06 | 3.03 (2791) |  | |  |
| Post-saccadic Direction × SAPP | -0.02 | 0.98 | 0.03 | 0.04 (457) |  | |  |
| Post-saccadic Distance × SAPP | -0.03 | 0.97 | 0.02 | 0.02 (557) |  | |  |
| Group × Target Displacement × Post-saccadic Direction |  |  |  | 0.36 (1834) |  | |  |
| Group × Target Displacement × Post-saccadic Distance |  |  |  | 0.94 (8928) |  | |  |
| Group × Target Displacement × SAPP |  |  |  | 0 (142) |  | |  |
| Group × Post-saccadic Direction × Post-saccadic Distance |  |  |  | 2.77 (2791) |  | |  |
| Group × Post-saccadic Direction × SAPP |  |  |  | 1.28 (457) |  | |  |
| Group × Post-saccadic Distance × SAPP |  |  |  | 5.29* (557) |  | |  |
| Post-saccadic Distance × Post-saccadic Direction × Target Displacement | -0.02 | 0.98 | 0.03 | 1.79 (10788) |  | |  |
| Target Displacement × Post-saccadic Direction × SAPP | 0.004 | 1.004 | 0.01 | 2.69 (3237) |  | |  |
| Target Displacement × Post-saccadic Distance × SAPP | -0.01 | 0.99 | 0.009 | 1.21 (10518) |  | |  |
| Post-saccadic Direction × Post-saccadic Distance × SAPP | -0.03 | 0.97 | 0.02 | 0.27 (3519) |  | |  |
| Group × Target Displacement × Post-saccadic Distance × Post-saccadic Direction |  |  |  | 0.25 (10788) |  | |  |
| Group × Target Displacement × Post-saccadic Direction × SAPP |  |  |  | 1.94 (3237) |  | |  |
| Group × Target Displacement × Post-saccadic Distance × SAPP |  |  |  | 5.94* (10518) |  | |  |
| Group × Post-saccadic Direction × Post-saccadic Distance × SAPP |  |  |  | 6.11* (3519) |  | |  |
| Post-saccadic Distance × Post-saccadic Direction × Target Displacement × SAPP | -0.01 | 0.99 | 0.009 | 0.22 (14114) |  | |  |
| Group × Target Displacement × Post-saccadic Distance × Post-saccadic Direction × SAPP |  |  |  | 2.34 (14114) |  | |  |
| Random Effects |  |  |  |  |  | |  |
| Intercept |  |  |  |  | | 1.47*** | 5.97 |
| Saccade Direction |  |  |  |  | | 0.19*** | 4.84 |
| Target Displacement |  |  |  |  | | 0.17*** | 4.91 |
| Post-saccadic Direction |  |  |  |  | | 0.005 | 0.38 |
| Post-saccadic Distance |  |  |  |  | | 0.01* | 1.77 |
| Saccade Direction × Target Displacement |  |  |  |  | | 0.007 | 0.98 |
| Saccade Direction × Post-saccadic Direction |  |  |  |  | | 0.03* | 2.17 |
| Saccade Direction × Post-saccadic Distance |  |  |  |  | | 0.004 | 1.25 |

**p* < .05, ***p* < .01, *** *p* < .001.

SAPP, Scale for the Assessment of Passivity Phenomena.

**Table S6.** Logistic multilevel regression coefficients estimating effects of group, disturbances in the subjective experience of self (measured by IPASE, with imputed means), target displacement, post-saccadic direction, and post-saccadic distance on perceptual judgment on the blanking task.

| Variable | *b* | Odds | Standard Error | *F(df)* | *σ^2^* | | *Wald Z* |
| --- | --- | --- | --- | --- | --- | --- | --- |
| Fixed Effects |  |  |  |  |  | |  |
| Intercept | 0.14 | 1.15 | 0.25 |  |  | |  |
| Group |  |  |  | 4.59* (136) |  | |  |
| Target Displacement | 1.44 | 4.22 | 0.11 | 267.08*** (150) |  | |  |
| Post-saccadic Direction | -0.04 | 0.96 | 0.10 | 4.91* (1131) |  | |  |
| Post-saccadic Distance | -0.03 | 0.97 | 0.07 | 3.39 (1849) |  | |  |
| IPASE | -0.003 | 0.997 | 0.005 | 1.49 (144) |  | |  |
| Group × Target Displacement |  |  |  | 0.11 (145) |  | |  |
| Group × Post-saccadic Direction |  |  |  | 1.51 (750) |  | |  |
| Group × Post-saccadic Distance |  |  |  | 1.47 (1199) |  | |  |
| Group × IPASE |  |  |  | 1.26 (133) |  | |  |
| Target Displacement × Post-saccadic Direction | 0.10 | 1.11 | 0.06 | 0.11 (6259) |  | |  |
| Target Displacement × Post-saccadic Distance | -0.03 | 0.97 | 0.04 | 3.49 (23757) |  | |  |
| Target Displacement × IPASE | 0.001 | 1.001 | 0.002 | 3.57 (155) |  | |  |
| Post-saccadic Direction × Post-saccadic Distance | 0.07 | 1.07 | 0.07 | 0 (15137) |  | |  |
| Post-saccadic Direction × IPASE | -0.002 | 0.998 | 0.002 | 0.34 (1108) |  | |  |
| Post-saccadic Distance × IPASE | -0.002 | 0.998 | 0.001 | 0.12 (1894) |  | |  |
| Group × Target Displacement × Post-saccadic Direction |  |  |  | 2.03 (5845) |  | |  |
| Group × Target Displacement × Post-saccadic Distance |  |  |  | 1.60 (18562) |  | |  |
| Group × Target Displacement × IPASE |  |  |  | 2.17 (142) |  | |  |
| Group × Post-saccadic Direction × Post-saccadic Distance |  |  |  | 1.83 (8252) |  | |  |
| Group × Post-saccadic Direction × IPASE |  |  |  | 0.94 (581) |  | |  |
| Group × Post-saccadic Distance × IPASE |  |  |  | 1.94 (998) |  | |  |
| Post-saccadic Distance × Post-saccadic Direction × Target Displacement | 0.03 | 1.03 | 0.04 | 0.04 (23757) |  | |  |
| Target Displacement × Post-saccadic Direction × IPASE | -0.001 | 0.999 | 0.001 | 0.21 (6571) |  | |  |
| Target Displacement × Post-saccadic Distance × IPASE | -0.0009 | 0.999 | 0.0006 | 0.13 (19112) |  | |  |
| Post-saccadic Direction × Post-saccadic Distance × IPASE | -0.002 | 0.998 | 0.001 | 0.05 (19662) |  | |  |
| Group × Target Displacement × Post-saccadic Distance × Post-saccadic Direction |  |  |  | 0.96 (22953) |  | |  |
| Group × Target Displacement × Post-saccadic Direction × IPASE |  |  |  | 0.83 (5584) |  | |  |
| Group × Target Displacement × Post-saccadic Distance × IPASE |  |  |  | 1.58 (13076) |  | |  |
| Group × Post-saccadic Direction × Post-saccadic Distance × IPASE |  |  |  | 1.39 (10457) |  | |  |
| Post-saccadic Distance × Post-saccadic Direction × Target Displacement × IPASE | -0.0008 | 0.999 | 0.0006 | 0.01 (23757) |  | |  |
| Group × Target Displacement × Post-saccadic Distance × Post-saccadic Direction × IPASE |  |  |  | 1.57 (19741) |  | |  |
| Random Effects |  |  |  |  |  | |  |
| Intercept |  |  |  |  | | 1.45*** | 6.73 |
| Saccade Direction |  |  |  |  | | 0.19*** | 5.49 |
| Target Displacement |  |  |  |  | | 0.23*** | 5.61 |
| Post-saccadic Direction |  |  |  |  | | 0.03* | 1.89 |
| Post-saccadic Distance |  |  |  |  | | 0.01* | 1.83 |
| Saccade Direction × Post-saccadic Direction |  |  |  |  | | 0.02* | 2.05 |
| Saccade Direction × Post-saccadic Distance |  |  |  |  | | 0.007* | 1.98 |

**p* < .05, ***p* < .01, *** *p* < .001.

IPASE, Inventory of Psychotic-like Anomalous Self-Experiences.

**Table S7.** Logistic multilevel regression coefficients estimating effects of group, target displacement, post-saccadic direction, and post-saccadic distance on perceptual judgment on the blanking task, breaking down by group.

| Variable | *b* | Odds | Standard Error | *F(df)* | *σ^2^* | *Wald Z* |
| --- | --- | --- | --- | --- | --- | --- |
| Fixed Effects |  |  |  |  |  |  |
| HC | -0.83 | 0.44 | 0.21 | 15.03*** (169) |  |  |
| SZ | -0.05 | 0.95 | 0.19 | 0.07 (145) |  |  |
| BPP | -0.79 | 0.45 | 0.21 | 14.01*** (131) |  |  |
| HC × Target Displacement | 1.83 | 6.23 | 0.11 | 295.70*** (239) |  |  |
| SZ × Target Displacement | 1.45 | 4.26 | 0.09 | 289.85*** (153) |  |  |
| BPP × Target Displacement | 1.52 | 4.57 | 0.10 | 244.21*** (141) |  |  |
| HC × Post-saccadic Direction | -0.28 | 0.76 | 0.10 | 7.32** (1200) |  |  |
| SZ × Post-saccadic Direction | -0.18 | 0.84 | 0.08 | 5.71* (543) |  |  |
| BPP × Post-saccadic Direction | -0.22 | 0.80 | 0.07 | 9.64** (221) |  |  |
| HC × Post-saccadic Distance | -0.11 | 0.90 | 0.07 | 2.38 (1945) |  |  |
| SZ × Post-saccadic Distance | -0.11 | 0.90 | 0.05 | 4.35* (902) |  |  |
| BPP × Post-saccadic Distance | -0.17 | 0.84 | 0.05 | 12.75*** (299) |  |  |
| HC × Target Displacement × Post-saccadic Direction | -0.11 | 0.90 | 0.06 | 2.76 (5527) |  |  |
| SZ × Target Displacement × Post-saccadic Direction | 0.03 | 1.03 | 0.04 | 0.64 (3433) |  |  |
| BPP × Target Displacement × Post-saccadic Direction | -0.03 | 0.97 | 0.05 | 0.34 (3911) |  |  |
| HC × Target Displacement × Post-saccadic Distance | -0.01 | 0.99 | 0.04 | 0.07 (10450) |  |  |
| SZ × Target Displacement × Post-saccadic Distance | -0.06 | 0.94 | 0.03 | 4.39* (7986) |  |  |
| BPP × Target Displacement × Post-saccadic Distance | -0.09 | 0.91 | 0.03 | 12.82*** (6875) |  |  |
| HC × Post-saccadic Direction × Post-saccadic Distance | -0.01 | 0.99 | 0.07 | 0.03 (14860) |  |  |
| SZ × Post-saccadic Direction × Post-saccadic Distance | -0.00004 | 0.99996 | 0.05 | 0 (5877) |  |  |
| BPP × Post-saccadic Direction × Post-saccadic Distance | -0.08 | 0.92 | 0.04 | 3.55 (2160) |  |  |
| HC × Target Displacement × Post-saccadic Distance × Post-saccadic Direction | 0.04 | 1.04 | 0.04 | 0.66 (13188) |  |  |
| SZ × Target Displacement × Post-saccadic Distance × Post-saccadic Direction | -0.004 | 0.996 | 0.03 | 0.03 (11412) |  |  |
| BPP × Target Displacement × Post-saccadic Distance × Post-saccadic Direction | -0.01 | 0.99 | 0.03 | 0.20 (8015) |  |  |
| Random Effects |  |  |  |  |  |  |
| Intercept |  |  |  |  | 1.46*** | 7.36 |
| Saccade Direction |  |  |  |  | 0.17*** | 5.76 |
| Target Displacement |  |  |  |  | 0.25*** | 6.19 |
| Post-saccadic Direction |  |  |  |  | 0.03* | 2.25 |
| Post-saccadic Distance |  |  |  |  | 0.01* | 1.92 |
| Saccade Direction × Target Displacement |  |  |  |  | 0.004 | 0.74 |
| Saccade Direction × Post-saccadic Direction |  |  |  |  | 0.04** | 2.76 |
| Saccade Direction × Post-saccadic Distance |  |  |  |  | 0.009* | 2.30 |

**p* < .05, ***p* < .01, *** *p* < .001.

**Figure S1.** Four-parameter (maximum value, minimum value, midway point between the maximum and minimum values, and slope) logistic fits of percentage of forward responses as a function of target displacement. Thicker lines represent fits of the group averages and thinner lines represent fits of individual participants.

**
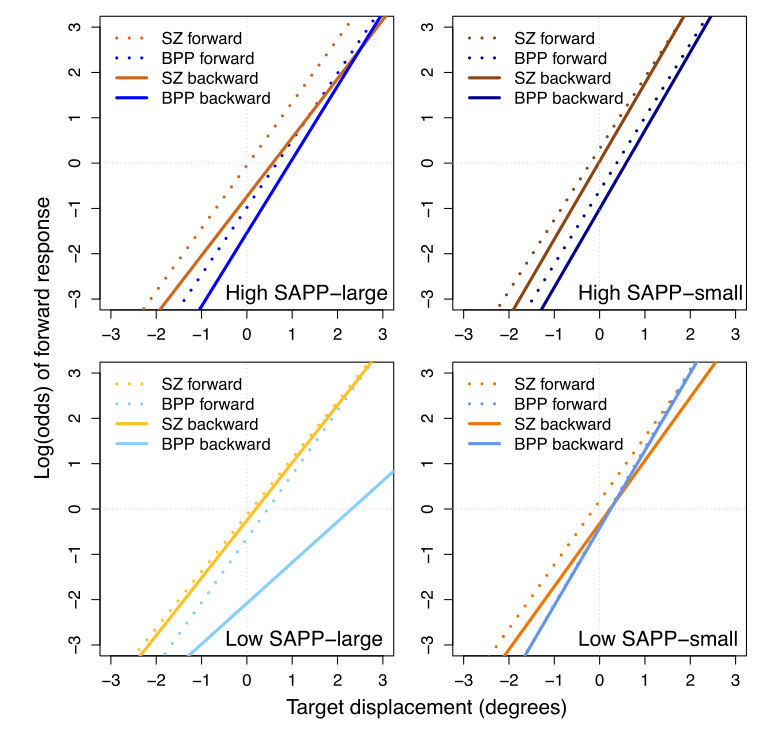
**

**Figure S2.** Lifetime Passivity symptoms x group x post-saccadic distance x post-saccadic direction interaction effect. The vertical axis represents the likelihood of making a forward response. Lines were plotted by computing a two-intercept model for each post-saccadic direction (forward vs. backward) and then calculating estimates based on large (1 SD above the mean) and small (1 SD below the mean) post-saccadic distance, and high (2.2 above the mean) and low (2.2 below the mean) SAPP score.

SAPP, Scale for the Assessment of Passivity Phenomena.

**Figure S3.** Delusions × post-saccadic direction interaction. The vertical axis represents the likelihood of making a forward response. Lines were plotted by computing a two-intercept model for each post-saccadic direction (forward vs. backward) and then calculating estimates based on large and small (1 SD above and below the mean) post-saccadic distance, and high and low (5 above and below the mean) delusion score.

**References**

1. Bono C, Ried LD, Kimberlin C, Vogel B. Missing data on the Center for Epidemiologic Studies Depression Scale: A comparison of 4 imputation techniques. *Res Soc Adm Pharm*. 2007;3(1):1-27. doi:10.1016/j.sapharm.2006.04.001

2. Downey RG, King CV. Missing Data in Likert Ratings: A Comparison of Replacement Methods. *J Gen Psychol*. 1998;125(2):175. doi:10.1080/00221309809595542

3. Hawthorne G, Hawthorne G, Elliott P. Imputing Cross-Sectional Missing Data: Comparison of Common Techniques. *Aust N Z J Psychiatry*. 2005;39(7):583-590. doi:10.1080/j.1440-1614.2005.01630.x

4. Cicero DC, Neis AM, Klaunig MJ, Trask CL. The Inventory of Psychotic-Like Anomalous Self-Experiences (IPASE): Development and validation. *Psychol Assess*. 2017;29(1):13-25. doi:10.1037/pas0000304

5. Yao B, Rolfs M, McLaughlin C, et al. Oculomotor corollary discharge signaling is related to repetitive behavior in children with autism spectrum disorder. *J Vis*. 2021;21(8):9. doi:10.1167/jov.21.8.9

6. Collins T, Rolfs M, Deubel H, Cavanagh P. Post-saccadic location judgments reveal remapping of saccade targets to non-foveal locations. *J Vis*. 2009;9(5):1-9. doi:10.1167/9.5.29

7. Hubbard TL. Forms of momentum across space: Representational, operational, and attentional. *Psychon Bull Rev*. 2014;21(6):1371-1403. doi:10.3758/s13423-014-0624-3

8. Rösler L, Rolfs M, van der Stigchel S, et al. Failure to use corollary discharge to remap visual target locations is associated with psychotic symptom severity in schizophrenia. *J Neurophysiol*. 2015;114(2):1129-1136. doi:10.1152/jn.00155.2015

9. Costa ALL, Costa DL, Pessoa VF, Caixeta FV, Maior RS. Systematic review of visual illusions in schizophrenia. *Schizophr Res*. 2023;252:13-22. doi:10.1016/j.schres.2022.12.030

1. We did not use 1 SD above and below the mean because 1 SD (2.73) is larger than the grand mean of SAPP (2.43). Therefore, we picked a value that is large enough to show the interaction effect but would not result in an invalid SAPP score. [↑](#footnote-ref-1)
2. We used 5 above and below the mean as high and low delusion scores. We did not use 1 SD above and below the mean because 1 SD (6.7) is larger than the grand mean of delusion (5.02). Therefore, we picked a value that is large enough to show the interaction effect but would not result in an invalid score. [↑](#footnote-ref-2)
